
JOURNAL INFORMATION
==============================
NLM Title Abbreviation: ScientificWorldJournal
Journal ID: ScientificWorldJournal
NLM Journal ID: 101131163
Journal ID: TSWJ

The Scientific World Journal

ISSN: 2356-6140
EISSN: 1537-744X
Publisher: Wiley

ARTICLE INFORMATION
==============================
PMCID: PMC6084210
PMID: 30147563
DOI: 10.1100/tsw.2001.203
Article ID: 937276
Article version: 1
Subjects: Short Report

Calpain Is a Major Regulator of Neutrophils Apoptosis

Altznauer Frank *
Simon Hans-Uwe
Department of Pharmacology, University of Bern, Friedbühlstr. 49, CH-3010 Bern, Switzerland
*Frank Altznauer: frank.altznauer@pki.unibe.ch
Publication date: 2001
Electronic publication date: 2001 Oct 12
Volume: 1
First page: 91
Last page: 91
Copyright: Copyright © 2001 Frank Altznauer and Hans-Uwe Simon.
Copyright year: 2001
License: This is an open access article distributed under the Creative Commons Attribution License, which permits unrestricted use, distribution, and reproduction in any medium, provided the original work is properly cited.
License URL: https://creativecommons.org/licenses/by/3.0/

Funding: Schweizerische Nationalfonds zur Förderung der Wissenschaftlichen Forschung 32-58916.99

==============================
Miami *Nature Biotechnology* Short Reports
*TheScientificWorld* (2001) 1 (S3), 91SR
ISSN 1532-2246; DOI 10.1100/tsw.2001.203

### CALPAIN IS A MAJOR REGULATOR OF NEUTROPHILS APOPTOSIS

Frank Altznauer* and Hans-Uwe Simon

Department of Pharmacology, University of Bern, Friedbühlstr. 49, CH-3010 Bern, Switzerland
* frank.altznauer@pki.unibe.ch

**INTRODUCTION.** Calpain is a calcium-activated neutral cysteine-protease, which exists in two isoforms: μ-calpain (lower calcium requirement) and m-calpain (higher calcium requirement). Both isoforms are ubiquitously expressed and exist as heterodimers of regulatory p30- and catalytic p80-subunits. Calpain has recently been shown to be involved in the onset of several neurological diseases, such as Alzheimer disease and multiple sclerosis (1,2). Calpain is activated in apoptotic cells via degradation of its endogenous inhibitor calpastatin, leading to degradation of the p30-subunit and autoproteolytic activation of the p80-subunit (3). There are several known proteolytic targets of the calpains, including fodrin, caspase-12, I-κB and bax.

In this report, we show that calpain is part of the CD95-mediated apoptotic pathway and cleaves bax into its proapoptotic form, which mediates breakdown of the mitochondrial potential and the activation of caspase-3.

**METHODS.** Neutrophils were isolated and cultured as previously described (4). For calpain inhibition, cells were pretreated with 50 μM E64d (Sigma) for 1h. Apoptosis-assays and immunoblotting were performed as previously described (4).

**RESULTS.** Spontaneous neutrophil apoptosis was associated with bax cleavage into an 18 kDa fragment (Fig A). FasR activation accelerated the cleavage of bax. The generation of the 18 kDa fragment and apoptosis were blocked by the specific calpain inhibitor E64d in the presence and absence of FasR activation (Fig. A, B). Calpain inhibition was also associated with maintenance of the mitochondrial potential (not presented) and inhibition of caspase-3 activation (Fig. C). To investigate the mechanism of calpain activation, we analyzed calpastatin levels by immunoblotting. Several calpastatin isoforms were observed in neutrophils and their levels appeared to correlate with the amount of apoptosis (Fig. D)

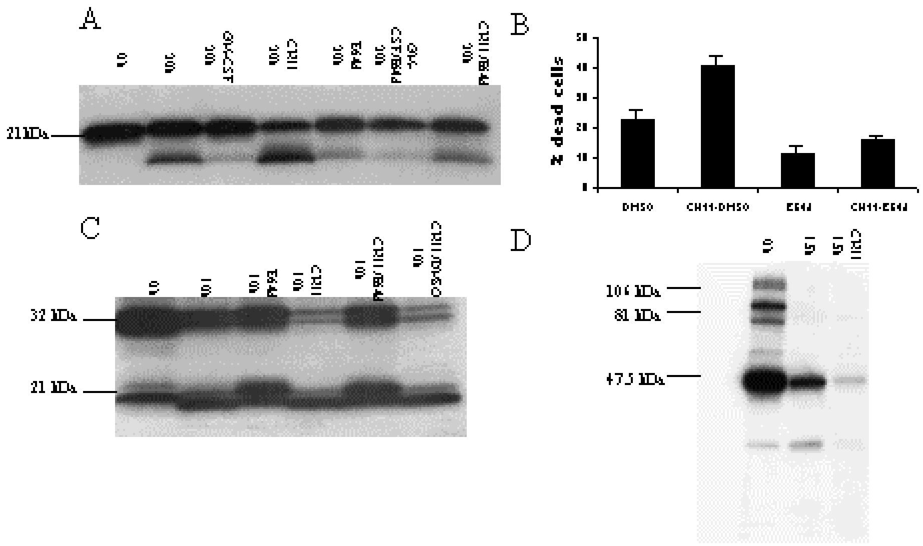

**DISCUSSION.** Calpain seems to be required for the conversion of p21 to p18 and appears to be a major player in spontaneous and FasR-mediated apoptotic pathways in neutrophils.

**ACKNOWLEDGEMENT.** This work was supported by the Swiss National Science Foundation (32-58916.99).

**REFERENCES.**
1. Shields, D.C., Schaecher, K.E., Saido, T.C., and Banik, N.L. (1999) Proc. Natl. Acad. Sci. U S A 96, 11486-11491
2. Lee, M., Kwon, Y.T., Li, M., Peng, J., Friedlander, R.M., and Tsai, L. (2000) Nature 405, 360-364
3. Squier, M.K.T., Sehnert, A.J., Sellins, K.S., Malkinson, A.M., Takano, E., and Cohen, J.J. (1999) J. Cell Physiol. 178, 311-319
4. Dibbert, B., Weber, M., Nikolaizik, W.H., Vogt, P., Schöni, M.H., Blaser, K., and Simon, H.U. (1999) Proc. Natl. Acad. Sci. U S A 96, 13330-13335
